# Validation of a criterion-based screening and triage pathway for adult ADHD: a prospective observational study of safety and operational efficiency

**DOI:** 10.3389/fpsyt.2026.1789686

**Published:** 2026-05-15

**Authors:** Marios Adamou, Tim Fullen, Rebecca Crisp, Emily Hallett, Lauren Spencer, Lydia Wharton, Sarah L. Jones

**Affiliations:** 1 University of Huddersfield, Huddersfield, United Kingdom; 2 School of Human and Health Sciences, South West Yorkshire Partnership Teaching NHS Foundation Trust, United Kingdom

**Keywords:** ADHD, ADHD diagnosis, adult ADHD, diagnostic assessment, service provision, triage

## Abstract

**Background:**

The increasing demand for adult attention-deficit hyperactivity disorder (ADHD) assessments has required the development of efficient triage pathways. This study provides a formal assessment of a criterion-based screening model designed to prioritise patient safety and operational efficiency within a National Health Service (NHS) specialist secondary care setting.

**Methods:**

A prospective observational validation design was employed, involving 49 consecutive adults referred for ADHD assessment none of whom had a previous ADHD diagnosis. The Comprehensive ADHD Screening Questionnaire (CASQ), a clinician-administered instrument based on DSM-5 criteria, was utilised by four trained Physician Assistants. To ensure an assessment of triage safety, a universal assessment model was adopted: all participants received a blinded, gold-standard diagnostic assessment (NICE-compliant) regardless of the initial triage recommendation thereby eliminating verification bias. The primary outcome measure was the Number Needed to Harm (NNH), defined as the number of people screened before a single false-negative result occurs.

**Results:**

Of the 48 participants who completed the diagnostic process, six (12.5%) received an ADHD diagnosis. The triage pathway correctly identified all six cases, resulting in a sensitivity of 100.0% (95% CI: 61.0%–100.0%) and an infinite NNH. Specificity was 45.2% (95% CI: 31.2%–59.9%), with a positive predictive value of 20.7%. The pathway permitted 39.6% (n = 19) of referrals to be triaged to alternative pathways rather than full ADHD assessment, potentially saving significant specialist clinician time. Exploratory analyses indicated that score magnitude did not reliably distinguish between true and false positives within the group triaged as appropriate for further assessment.

**Conclusions:**

These preliminary findings suggest that criterion-based screening conducted by appropriately trained non-specialist clinicians can achieve high levels of safety whilst improving service efficiency. The findings support the feasibility of task-shifting models in adult ADHD services, provided that triage thresholds are calibrated to prioritise sensitivity. These results require replication in adequately powered multi-site studies before firm conclusions regarding pathway safety can be drawn. Further research is required to establish inter-rater reliability and cost-effectiveness across diverse clinical settings.

## 1 Introduction

Adult attention-deficit hyperactivity disorder (ADHD) has transitioned from relative clinical obscurity to become one of the most frequently requested assessments in contemporary mental health services. Historically conceptualised primarily as a childhood condition, evidence demonstrates that ADHD persists into adulthood in approximately 50-60% of cases (1) with global prevalence estimates ranging from 2.5% to 5% in adult populations (2). This recognition has coincided with substantial increases in self-referrals and clinical demand, creating pressures on mental health services where waiting times for assessment often extend to months or even years (3), contributing to a diagnostic bottleneck that hinders timely access to appropriate care and support (4). Such delays not only affect individual wellbeing but also strain overall service provision (5), leading to broader implications for resource allocation within healthcare systems.

The clinical consequences of this bottleneck are important because adults with unrecognised ADHD frequently present with functional impairment across occupational, educational, and interpersonal domains (6), alongside increased risks of accidents (7) substance misuse (8), and comorbid psychiatric conditions such as anxiety or depression (9). The historical failure to recognise and treat ADHD symptoms across previous decades has resulted in a missing middle generation of adults who were never identified during childhood and now present with complex, long-standing difficulties that have accumulated over time (10). Additionally, rising public awareness of ADHD through social media platforms and online communities has generated additional screening volume, with increasing numbers of adults seeking assessment based on self-identification (11) or social media descriptions (12, 13) potentially reducing the proportion of true cases amongst those presenting to services and for a variety of factors also introduce a risk of overdiagnosis (14, 15). This surge in awareness, whilst beneficial in reducing stigma, also introduces challenges in distinguishing between genuine neurodevelopmental concerns and transient or situational difficulties.

The convergence of increased demand, limited specialist capacity, and heterogeneous severity of presenting cases has prompted services to explore structured screening and triage as potential solutions to manage referral flow and prioritise clinical need. Such approaches aim to differentiate between people requiring specialist neurodevelopmental assessment and those whose difficulties may be better addressed through alternative pathways or interventions.

### 1.1 Screening and triage pathways in healthcare

Screening and triage pathways are not uncommon in healthcare and especially across emergency, urgent, and primary care, structured clinical triage has been found to improve timeliness, flow, and workload management, and generally supports safe care, especially when combined with training or enhanced staffing models. However, evidence that triage systems on their own improve mortality and other hard clinical outcomes is still limited, and under triage remains a safety concern, particularly for complex conditions and vulnerable groups (16–19). Across mental health settings, triage models are associated with better prioritisation, timeliness, use of alternatives to admission, and staff confidence, and they can support safer crisis responses. However, high quality evidence directly linking mental health triage to improved long term clinical outcomes, reduced harm, or cost effectiveness remains limited, and variability in implementation are important ongoing risks (18, 20–24). For broader neurodevelopmental referrals and mainly Autism, structured triage has early evidence of safely reducing inappropriate assessments, shortening waits, improving satisfaction, and potentially saving costs, with AI supported triage showing promise but not yet routine (25–28). There is no direct research addressing this issue in the context of ADHD. Although one study recommends developing a range of referral pathways based on case complexity (29) and another highlights the need for greater training amongst clinical staff, particularly general practitioners and adult mental health services (30), the evidence base remains limited. From our review of the literature, there is no clinical triage model adult ADHD.

This absence may reflect inherent challenges in applying symptom-based triage to ADHD, given the extensive symptom overlap between its core symptoms and mood, anxiety, bipolar and substance use disorders, driven by shared features such as inattention, restlessness and emotional dysregulation (9, 31–44). When symptoms do not have sufficient discriminatory power to differentiate between conditions, operationalizing a clinical model becomes a challenge as the reliability of symptom-based triage decisions becomes questionable, potentially leading to both under-triage of genuine ADHD cases and inappropriate referral of people whose difficulties primarily reflect alternative diagnoses. Given the limitations of symptom-based triage for ADHD, services have traditionally predominantly focussed on screening approaches rather than clinical triage models, employing standardised self-report instruments.

### 1.2 Current screening approaches in adult ADHD

Traditional ADHD assessment in adults relies heavily on retrospective self-report of childhood symptoms and current functioning, methods that are vulnerable to recall bias, response bias, and symptom exaggeration or minimisation (45). Self-rating scales, whilst efficient and widely used, demonstrate poor discriminant validity in differentiating ADHD from other psychiatric conditions (46).

The majority of contemporary screening pathways employ brief self-report instruments with the Adult ADHD Self-Report Scale (ASRS) representing the most widely validated and implemented tool across various clinical settings (46, 47). Developed in collaboration with the World Health Organisation, the ASRS captures ADHD symptoms addressing limitations of earlier tools focussed on childhood (48). The instrument exists in an 18-item full version and a 6-item screener (ASRS-v1.1), with the shorter version typically employed for initial screening due to its practicality in high-volume services (49). The updated ASRS-5 aligns with DSM-5 criteria and claims increased capacity to capture nuanced adult presentations whilst incorporating developmental and situational context (50). Validation studies of the ASRS in selected populations demonstrate its high sensitivity (up to 91%) and specificity (74-96%), with area under the curve values up to 0.94, indicating strong discriminative performance (51, 52). The tool has been adapted for use in other languages including versions in Japanese and French which have confirmed its validity across different cultures supporting its global applicability (53, 54).

A systematic review and meta-analysis of screening tools for adult ADHD in primary care found that the ASRS-6 (DSM-5 version) had the highest test accuracy, with pooled sensitivity of 0.83 (95% CI: 0.67-0.92), specificity of 0.87 (95% CI: 0.80-0.93), area under the curve of 0.92, and low heterogeneity (52). The brief administration time of the ASRS-v1.1 (average 54.3 seconds) makes it particularly suitable for screening in busy primary care practices (47). Studies examining feasibility and acceptability amongst general practitioners have assessed the tool positively, though potential problems were raised regarding treatment options, stigmatisation, and knowledge gaps amongst primary care physicians (55).

However, studies show substantial variation in performance across different clinical populations. Whilst the ASRS maintains high sensitivity in general population samples, specificity reduces considerably in clinical samples with symptoms characteristic of other psychiatric disorders, due to symptom overlap with conditions such as anxiety, depression, and substance use disorders, which can mimic ADHD features (56, 57). Van de Glind et al. (58) reported acceptable sensitivity (0.84) but lower specificity (0.66) when the ASRS was administered to patients with substance use disorders. Similarly, Hines, King and Curry (47) found strong sensitivity (1.0) but weaker specificity (0.71) in primary care patients. In adults with Major Depressive Disorder, Dunlop, Wu and Helms (59) observed lower sensitivity (0.60) and specificity (0.69). These findings therefore suggest that the performance of ASRS varies substantially across contexts, particularly in populations with high comorbidity, where symptom overlap reduces specificity and increases the likelihood of false-positive results.

### 1.3 Alternative screening instruments

The Conners’ Adult ADHD Rating Scales brief self-report ADHD Index (CAARS ADHD Index) is another validated option (60). However, due to its cost and requirement for norm-referenced scoring, the CAARS ADHD Index may prove burdensome for routine use by many clinicians. The CAARS has demonstrated good psychometric properties across diverse populations. Machine learning studies using all 26 items of the CAARS achieved 80% global accuracy in multiclass classification of ADHD versus other conditions, with precision ranging from 0.78 to 0.92 and sensitivity from 0.58 to 0.87 for ADHD (61). The CAARS includes embedded validity indicators, including the Infrequency Index and ADHD Credibility Index, designed to detect invalid responding and symptom exaggeration (62). Research on combining multiple validity indicators has shown that a combined index had sensitivity of 41-51% and specificity above 87% for detecting simulators, which may be particularly important in forensic, disability, and accommodation-seeking contexts where secondary gain may influence responding (62, 100).

In exploring other tools, a comparative analysis of screening instruments has shown that their performance and methodology differ. For example, the Wender Utah Rating Scale assesses retrospective childhood symptoms effectively (63), whilst the ASRS captures current symptoms in adulthood (52). Instruments such as TRAQ10 (51), and ASSET-BS (64) offer alternatives with distinct advantages in specific clinical contexts, allowing for some choice in approach (64). Validation studies in substance use disorder populations, where ADHD is highly prevalent yet frequently goes unrecognised, found that the WURS, CAARS, and ASRS-v1.1 all demonstrated adequate sensitivity, specificity, and predictive values when validated against structured diagnostic interviews in outpatient cocaine dependence treatment settings (65). This suggests that rating scales retain utility even in populations with high rates of comorbidity, though clinicians must remain vigilant about symptom misattribution (65). A systematic review concluded that ASRS-6 (DSM-5), WURS-25, CAARS-s:sV, and TRAQ10 are suitable instruments for screening in primary care settings, though feasibility studies are needed to provide more evidence for implementation of WURS-25 and CAARS-s:sV in primary care (52). Recently, to bring the whole field together, an analysis of 40 tools found no association between cost and diagnostic accuracy, with brief, freely available self-report tools performing comparably to longer or commercially available alternatives (66). For all the screening tools, clinical utility is constrained by their predictive values. The ASRS for example has high negative predictive values (exceeding 95%), making it effective for ruling out ADHD and preventing unnecessary assessments (66). However, positive predictive values range from 10% to 30% at population base rates, indicating that most positive screens do not result in ADHD diagnoses following full assessment (66, 67).

### 1.4 Limitations of self-report screening

Self report questionnaires however have well known limitations across many conditions, and ADHD is no exception (68). In ADHD specifically, these tools focus on current symptoms and, because of this, they don’t align with the full DSM 5 diagnostic criteria. They typically don’t evaluate whether symptoms were present before age 12, whether they occur across multiple settings, whether they cause functional impairment, or whether alternative explanations have been ruled out. This is why people with attentional, impulsive or hyperactive difficulties secondary to depression, anxiety, or stress may report items on ADHD screening tools without these being due to ADHD, whilst those with ADHD and well-developed compensatory strategies may underreport symptoms due to habituation or lack of awareness (69). Compounding this, retrospective childhood assessment also presents challenges due to poor recall accuracy and the frequent absence of collateral information from family or educators (70). Validity reduces in populations with substance use disorders through underreporting, potentially linked to impaired insight or competing priorities (71), in females with predominantly inattentive presentations that may be less overt (72, 73), and across cultural contexts where symptom interpretation varies (74). Symptom overlap leads to diagnostic overshadowing, where attention difficulties are attributed to more prominent mood disorders (38), and comorbidity reduces specificity in diverse clinical samples (75, 76). These limitations suggest the need for alternative screening approaches that more directly operationalize DSM-5 diagnostic criteria.

### 1.5 Digital assessment tools and continuous performance tests

Beyond self-report questionnaires, continuous performance tests (CPTs) represent a class of computerised cognitive assessments designed to measure sustained attention, response inhibition, and reaction time variability, the cognitive functions commonly impaired in ADHD (77). These tools aim to provide objective, quantifiable measures that are standardised across administrations and less susceptible to subjective bias than self-report measures (78).

The Quantified Behaviour Test (QbTest) is the most extensively researched digital assessment tool for ADHD (79). The QbTest combines a traditional continuous performance test with infrared motion tracking technology to simultaneously measure the three core ADHD symptom domains: inattention, impulsivity, and hyperactivity ( (80). During the 15–20-minute test, participants wear a headband with an infrared reflector whilst responding to visual stimuli presented on a computer screen, with the motion tracking system recording head movements in three-dimensional space to provide an objective measure of motor activity (80). The QbTest generates three primary scores of QbInattention, QbImpulsivity, and QbActivity which are standardised against age- and sex-matched normative data and combined into a composite QbTotal score (81).

The MOXO-d CPT is another commercially available continuous performance test, distinguished by including visual and auditory distractors to create a more ecologically valid assessment of attention under conditions that simulate real-world demands (82). This design is based on the premise that people with ADHD show particular difficulty maintaining attention in the presence of environmental distractors, a common real-world challenge (82). Other established CPT platforms include the Test of Variables of Attention (TOVA) (83), Conners’ Continuous Performance Test (CPT-II and CPT-3) (84), and the Integrated Visual and Auditory Continuous Performance Test (IVA+Plus) (85), though these have been less extensively validated in adult populations (86).

### 1.6 Diagnostic accuracy and clinical utility of digital tools

Studies examining the diagnostic accuracy of CPTs have produced mixed findings. The QbTest has moderate diagnostic accuracy when differentiating adults with ADHD from healthy controls, with sensitivity ranging from 83% to 91% and specificity from 57% to 85% depending on the comparison group (80). However, specificity reduces dramatically when comparing ADHD to other psychiatric conditions with overlapping symptoms, falling to 36-41% in some studies (87). This pattern reflects the transdiagnostic nature of attention and executive function deficits, which occur across multiple psychiatric and neurological conditions (88).

Research examining the incremental validity of CPTs beyond rating scales has shown that adding QbTest to clinical rating scales can improve classification accuracy from 81% to 90% when differentiating adult ADHD from autism spectrum disorder (89). The best evidence for clinical utility comes from a randomised controlled trial involving 250 children and young people, which found that clinicians with access to QbTest reports were significantly more likely to reach a diagnostic decision (hazard ratio 1.44), with QbTest reducing appointment length by 15% and increasing clinicians’ confidence in their diagnostic decisions without compromising diagnostic accuracy (90). These findings suggest that CPTs may increase the efficiency of ADHD assessment pathways, allowing greater patient throughput with clinicians reaching diagnostic decisions faster (90).

### 1.7 Limitations of digital assessment tools

Despite these potential benefits, CPTs have several limitations that constrain their utility as screening instruments. Multiple studies emphasise that CPTs show limited discriminant validity in differentiating ADHD from other psychiatric conditions with overlapping symptoms (87, 91). When used as standalone measures, CPTs demonstrate modest diagnostic accuracy with area under the receiver operating characteristic curve values typically ranging from 0.61 to 0.87, which is insufficient for definitive diagnosis without additional clinical information (89, 91).

Furthermore, most CPT research has focussed on paediatric populations, with relatively limited validation in adult samples (79, 86). The cost and resource requirements of CPTs including equipment purchase, software licensing, technical support, and staff training present practical barriers to implementation, with health economic analyses suggesting that whilst CPTs improve efficiency, cost savings remain modest (90, 92). Additionally, traditional CPTs are administered in quiet, distraction-free environments that do not reflect the complex, multitasking demands of real-world functioning, potentially limiting their ecological validity (82). Studies have found only modest correlations between CPT performance and self-reported symptoms, raising questions about what CPTs are measuring and whether they capture the subjective experience of ADHD (81).

### 1.8 Alternative approaches to self-report tools

An alternative to self report tools or digital assessment tools and continuous performance test would be a direct clinical assessment of DSM 5 diagnostic criteria using structured interviews such as the Diagnostic Interview for ADHD in Adults (DIVA). Although this method is not, on its own, sufficient to establish a diagnosis (93), it does allow for a systematic exploration of childhood symptoms, current symptoms, and functional impairment across life domains (94). A triage model based on such interviews would operationalize diagnostic requirements more precisely, offering stronger discriminative validity than tools focussed solely on self-reporting of symptoms (95). However, this approach may be viewed as extending beyond screening and triage, as it moves into the territory of diagnostic assessment.

As an alternative to traditional screening methods, criterion based interviews would occupy an intermediate position between self report questionnaires and full diagnostic assessment. Self report instruments capture recent symptom frequency from the individual’s perspective, whereas semi structured diagnostic interviews provide a detailed, systematic evaluation of DSM 5 criteria alongside developmental history. Criterion based screening, by contrast, assesses whether an individual is likely to provide diagnostically meaningful responses during a full assessment. This enables clinicians to identify cases where a comprehensive diagnostic assessment is likely to produce positive findings.

It is important to distinguish the present approach from conventional screening tool validation. The instrument under investigation is not a self-report questionnaire requiring psychometric characterisation of a latent construct but it is a clinician-administered structured pathway that operationalizes DSM-5 diagnostic criteria within a time-limited clinical encounter. The appropriate validation for such a pathway is criterion validation against diagnostic outcomes, which is the purpose of the present study.

Despite the theoretical advantages of criterion based screening and triage in ADHD, empirical evidence on the safety and effectiveness of this approach remains limited, underscoring the need for further research. Several questions remain unresolved. First, can clinicians reliably apply DSM 5 criteria within time limited screening contexts, or does accurate criterion based assessment require specialist expertise typically found only in tertiary services? Primary care providers frequently report limited confidence in managing complex ADHD presentations, raising concerns about their ability to conduct accurate criterion based screening without additional training (96). Second, what threshold should determine progression to a full assessment within criterion based pathways? Should people be referred if they potentially meet any DSM 5 criterion, if they meet a minimum number of criteria, or only if they appear to meet full diagnostic criteria? This decision has major implications for pathway sensitivity and specificity: more inclusive thresholds maximise safety but risk generating excessive demand for specialist assessments and straining service capacity.

Third, how do criterion based pathways compare with self report screening tools in terms of diagnostic accuracy across diverse populations? Although structured diagnostic interviews show stronger adherence to DSM 5 criteria, it remains uncertain whether brief criterion based screening interviews conducted by non-specialists can achieve acceptable sensitivity and specificity without compromising equity.

Fourth, what are the resource implications of implementing criterion based pathways across different healthcare systems? If such interviews require substantially more clinician time than self report questionnaires which patients can complete independently their adoption may require significant service redesign, workforce development, and resource allocation to ensure feasibility (49).

### 1.9 The present study

The primary aim of this study was to address these gaps of criterion-based screening and triage in ADHD via conducting a formal validation of the safety and feasibility of a criterion-based screening and triage pathway implemented by non-specialist clinicians. Specifically, we sought to determine whether such a pathway could reliably identify people requiring specialist ADHD assessment without risk of inappropriate exclusion (sensitivity), whilst also exploring its potential to improve service efficiency through appropriate screening-out of referrals unlikely to meet diagnostic criteria (specificity). To ensure rigorous assessment of pathway safety, all participants received comprehensive NICE-compliant diagnostic assessment regardless of their initial triage recommendation, thereby permitting identification of any false-negative results that would otherwise remain undetected in routine clinical practice.

## 2 Methods

### 2.1 Study design and setting

This study employed a prospective observational validation design to conduct a formal assessment of a criterion-based screening and triage pathway for adult attention-deficit hyperactivity disorder (ADHD). The evaluation was conducted within the specialist secondary care services at South West Yorkshire Partnership NHS Foundation Trust from January 2024 to February 2025. To address the critical clinical problem of diagnostic uncertainty and potential delays in definitive treatment, this study utilised a universal assessment model wherein all participants received comprehensive National Institute for Health and Care Excellence (NICE)-compliant diagnostic assessment regardless of initial triage recommendation. This methodology, inspired by the lived experience of patients and relatives, permitted rigorous assessment of triage safety by identifying any potential false-negative results that would typically remain undetected in routine clinical practice. The universal assessment model also eliminated verification (work-up) bias, as the diagnostic reference standard was applied to all participants irrespective of triage classification.

### 2.2 Participants and sampling

The study cohort consisted of 49 consecutive adults referred for specialist ADHD assessment during the evaluation period. All participants were undergoing their first specialist ADHD assessment; none had a prior ADHD diagnosis. All were referred through standard NHS pathways following self-referral or clinician referral for suspected ADHD. To maximise the ecological validity of the findings and reflect a real-world clinical population, inclusion criteria required participants to be aged 18 years or older, possess no known intellectual disability, and demonstrate the capacity to engage with the multi-stage diagnostic process. This convenience sample comprised the next 49 consecutive cases on the waiting list and reflected routine clinical practice, representing the typical case mix presenting to the service. Of the 49 people initially screened, one participant was excluded from the final statistical analysis due to relocation prior to completion of the diagnostic process, which rendered their final clinical outcome undetermined. Consequently, the final analytical sample comprised 48 participants. Sample size was determined by service capacity constraints and consecutive referral flow during the evaluation period rather than formal statistical power calculations.

To enable rigorous assessment of triage safety, all 49 cases received a full diagnostic assessment in accordance with the NICE guideline NG87 regardless of the triage decision. This design enabled direct comparison between triage recommendations and gold-standard diagnostic outcomes, thereby permitting calculation of sensitivity, specificity, and number needed to harm for the triage process. In routine clinical practice, people triaged as not appropriate for comprehensive assessment would not proceed to full diagnostic assessment; however, for evaluation purposes, universal comprehensive assessment was undertaken.

### 2.3 The criterion-based assessment instrument

#### 2.3.1 Overview and structure

The primary screening instrument under investigation was the Comprehensive ADHD Screening Questionnaire (CASQ), a study-specific inventory developed by the first author to systematically examine DSM-5 diagnostic criteria. The CASQ was designed for feasibility within a 60-minute clinical encounter, consisting of a 45-minute face-to-face interview followed by 15 minutes of administrative synthesis. The CASQ is organised into multiple sections designed to capture information relevant to each diagnostic criterion whilst remaining feasible for completion within a single clinical encounter.

To clarify the current validation status of each CASQ component, the criterion-based scoring algorithm (the primary determinant of triage decisions) is validated in the present study against diagnostic outcomes; the observational rating scales (Inattention and Hyperactivity-Impulsivity subscales) have face validity but have not yet undergone formal inter-rater reliability testing, which is the subject of a separate study; the functional impairment rating scale is under development and will be presented in a forthcoming publication.

The complete Comprehensive ADHD Screening Questionnaire (CASQ) template is not publicly available as several components are undergoing formal psychometric validation through separate research programmes. The functional impairment rating scale will be presented in a forthcoming publication, whilst the observational rating scales have not yet undergone inter-rater reliability testing. The instrument also has potential commercial applications that require consideration prior to public release. The present manuscript provides sufficient methodological detail to permit critical appraisal of the study design and findings, including operational definitions for each criterion assessment, specific rating scale items with behavioural anchors, and the complete decision-making algorithm for triage recommendations.

##### 2.3.1.1 Section 1: referral administration

This section captures demographic information including full name, preferred name, date of birth, gender and preferred pronouns, NHS number, and contact details with preferred communication mode. Administrative screening questions address several domains: the presence of diagnosed learning disability (which would necessitate referral to specialist learning disability services); consent to referral and clinical records review; communication needs requiring assessment adaptations; and practical considerations that might complicate clinic attendance.

##### 2.3.1.2 Section 2: relevant history

This section gathers contextual information through structured questions addressing pending medical, mental health, or developmental assessments or investigations; current or previous treatment for physical or mental health conditions; risks identified at the time of referral; past or current contact with the criminal justice system; and anticipated changes in circumstances that might affect assessment timing or engagement.

##### 2.3.1.3 Section 3: criterion-based assessment

This section systematically addresses each DSM-5 diagnostic criterion through a combination of structured questions and clinician observation.

###### 2.3.1.3.1 Assessment of DSM-5 criterion A: presence of ADHD symptoms

To address the requirements of the DSM-5 to assess pervasiveness and impairment, the assessment of criterion A utilises a bespoke mental-state inventory designed to capture both typical and atypical behavioural features. Rather than relying on traditional self-report methods, clinicians employed two structured observation scales: an Inattention subscale (five items) and a Hyperactivity-Impulsivity subscale (seven items). Each item was operationalized as a granular, observable behaviour feasible for rating during a single assessment, such as motor restlessness, distractibility, or intrusive speech.

The observational rating items were derived directly from DSM-5 symptom descriptions, operationalized to capture behaviours observable during a clinical interview. Each item represents a pragmatic translation of DSM-5 symptoms into observable behaviours feasible to rate during a 45-minute screening appointment. They are in effect prompts linked to the core symptoms and map directly onto DSM-5 diagnostic criteria, providing face validity for the assessment approach.

The Inattention subscale has five items derived from DSM-5 inattention criteria: (1) Does not seem to listen when spoken to directly, operationalized as maintaining eye contact and responding appropriately to questions; (2) Appears easily distracted by extraneous stimuli, assessed through observation of reactions to environmental noise or movement; (3) Has difficulty staying focussed during the assessment conversation, examined through ability to remain on topic without redirection; (4) Appears forgetful during the conversation, assessed through recall of earlier discussion points; and (5) Struggles to stay on topic during conversation, operationalized as frequency of deviation requiring clinician redirection.

The Hyperactivity-Impulsivity subscale has seven items: (1) Fidgets with or taps hands or feet, or squirms in seat; (2) Appears restless and unable to remain seated for extended periods; (3) Is “on the go”, appears as if “driven by a motor”; (4) Talks excessively; (5) Blurts out responses or interrupts during conversation; (6) Has difficulty waiting or pausing appropriately during conversation; and (7) Interrupts or intrudes on others during the session.

Ratings were recorded on a 5-point Likert scale with detailed behavioural anchors to ensure consistency and face validity: 0 (Never), 1 (Rarely), 2 (Sometimes), 3 (Often), and 4 (Very Often). For example, for the item “Does not seem to listen when spoken to directly”, the anchors are: 0 = “Patient always maintains eye contact and responds appropriately”; 1 = “Patient occasionally breaks eye contact but generally responds correctly”; 2 = “Patient frequently looks away, needs some prompting to refocus on questions”; 3 = “Patient often seems disengaged, needing frequent reminders to respond”; 4 = “Patient consistently appears not to listen, even with direct prompting”.

The Inattention subscale produces a total score ranging from 0-20, whilst the Hyperactivity-Impulsivity subscale produces a total score ranging from 0-28. These total scores, combined with clinical judgement regarding the quality and specificity of observed behaviours, informed the assessment of criterion A.

###### 2.3.1.3.2 Assessment of DSM-5 criterion B: age of onset

Criterion B requires that several inattentive or hyperactive-impulsive symptoms were present before age 12 years. Retrospective accounts were gathered to identify the presence of symptoms before age 12 through two open-ended questions or versions of: “At what age did you first notice symptoms that might be related to ADHD?” and “Describe the early symptoms and any difficulties you had in childhood or adolescence.” These questions enable people to provide retrospective accounts in their own words, which the clinician evaluates for consistency with DSM-5 symptom descriptions and appropriate developmental timing. The clinician exercises clinical judgement and asks expanding questions to determine whether the person’s account provides adequate evidence of childhood onset, accounting for common difficulties with precise retrospective recall.

###### 2.3.1.3.3 Assessment of DSM-5 criteria C and D: impairment across settings

Criterion C requires that symptoms are present in two or more settings (for example, at work and at home, at home and with friends), whilst criterion D requires clear evidence that symptoms interfere with or reduce the quality of social, academic, or occupational functioning. These criteria are assessed through a bespoke functional impairment rating scale across five life domains: Work/Education, Relationships/Family, Social Contacts, Free Time/Hobbies, and Self-Confidence/Self-Image.

The functional impairment scale employed in this study is currently under development and will be presented in detail in a separate publication. For the purposes of the present evaluation, the scale demonstrated adequate face validity and clinical utility for operationalizing DSM-5 Criteria C and D within the screening context. Each domain is rated using structured criteria with detailed behavioural anchors to guide clinician ratings. The scale yields both individual domain scores and a Total Functional Impairment Score, from which an Overall Impairment Level is derived.

During the screening interview, clinicians conduct systematic discussion of functioning in each domain, gathering specific examples of difficulties and their impact on daily life. Functional impairment ratings are completed during the administrative time following the face-to-face interview, allowing the clinician to integrate information gathered throughout the assessment. For triage purposes, criterion C (impairment across multiple settings) is considered met when difficulties are documented in at least two life domains, whilst criterion D (functional impact) is considered met when the Overall Impairment Level reaches a predetermined threshold indicating clinically significant impairment.

###### 2.3.1.3.4 Assessment of DSM-5 criterion E: exclusion of other mental disorders

Criterion E requires that symptoms do not occur exclusively during the course of schizophrenia or another psychotic disorder and are not better explained by another mental disorder. A dedicated section prompted systematic consideration of alternative diagnoses to ensure symptoms were not better explained by mood disorders, anxiety, or situational stressors.

During the Relevant History section, clinicians document: any diagnosed mental health conditions and treatments received; other health issues or concerns potentially related to the symptoms; previous psychological assessments including outcomes and recommendations; and family history regarding ADHD or other mental health conditions.

Additional open-ended questions explore coping strategies or self-management techniques currently used; and whether there have been periods where symptoms were absent or significantly reduced, with description of circumstances. These questions assist in differentiating neurodevelopmental ADHD (characterised by persistent, pervasive symptoms from childhood) from attention difficulties secondary to mood disorders, psychosis, or situational stressors (which typically show greater temporal variability and relationship to specific triggers or episodes).

The clinician exercises clinical judgement to determine whether reported attention difficulties are better accounted for by depression, anxiety, trauma-related conditions, substance use, psychotic disorders, personality disorders, or other psychiatric presentations. Where significant diagnostic uncertainty exists due to complex comorbidity, the individual would typically be referred for comprehensive assessment to enable more detailed differential diagnosis.

### 2.4 Clinical administration and triage logic

#### 2.4.1 Clinician training and qualifications

Screening assessments were conducted by a team of Physician Assistants with extensive specialist experience in adult ADHD. The screening clinicians received standardised training in the use of the CASQ comprising of didactic teaching on DSM-5 diagnostic criteria for ADHD; instruction on the structure and content of the CASQ; practice in applying the observational rating scales and functional impairment ratings; discussion of differential diagnosis considerations; and observed practice interviews with feedback from experienced assessors. Training emphasised calibration of observational ratings through joint scoring of video-recorded interviews, discussion of discrepant ratings, and consensus exercises to ensure consistent application of behavioural anchors across all four screening clinicians. Formal inter-rater reliability coefficients for the observational subscales are not yet available and will be reported in a dedicated psychometric study.

Training emphasised the importance of distinguishing ADHD from conditions with overlapping symptomatology, particularly mood and anxiety disorders. The clinician was trained to attend to developmental history, pattern of symptom persistence across time and contexts, and the quality of symptom descriptions provided by people. The training programme comprised four hours of instruction and required successful completion of ten supervised practice assessments before independent screening was permitted.

#### 2.4.2 Interview structure and duration

Screening assessments comprised a 45-minute face-to-face clinical interview followed by 15 minutes of clinician administrative time to complete the assessment documentation, totalling 60 minutes per case. This structure provided sufficient time for systematic examination of all assessment components whilst remaining practical within routine clinical services.

The face-to-face interview component typically followed this structure: (1) Introduction and consent confirmation (5 minutes) - clinician introduces the purpose of the screening interview, confirms consent to the assessment, and establishes rapport; (2) Administrative and contextual information gathering (10 minutes) - completion of referral administration and relevant history sections, including clarification of pending assessments, current treatments, and practical considerations; (3) Exploration of the individual’s perspective and expectations (5 minutes) - discussion of why the individual believes they have ADHD, hoped-for outcomes from assessment, and anticipated impact of diagnosis; (4) Assessment of childhood onset (5 minutes) - systematic exploration of early symptom onset, childhood experiences, and developmental history; (5) Assessment of current functional impairment (10 minutes) - detailed discussion of impact across work/education, relationships/family, social contacts, hobbies, and self-confidence domains; (6) Differential diagnosis exploration (5 minutes) - discussion of mental health history, alternative explanations for symptoms, and periods of symptom fluctuation; (7) Conclusion and explanation of next steps (5 minutes) - clinician summarises the screening discussion and outlines subsequent assessment procedures.

Following the face-to-face interview, the clinician used 15 minutes of administrative time to complete the criterion A observation ratings based on behaviours observed throughout the interview, finalise functional impairment ratings, complete criterion scoring, determine triage decision, and document clinical reasoning.

This structure was presented as guidance rather than rigid prescription, allowing the clinician to adapt the sequence and time allocation based on individual presentation and clinical need. The observational component of criterion A assessment occurred continuously throughout the interview rather than being restricted to specific time periods.

#### 2.4.3 Decision-making threshold for referral to full diagnostic assessment

The triage decision-making logic was intentionally calibrated to ensure safety whilst maintaining operational efficiency in a capacity-constrained service. Following completion of the CASQ, the clinician synthesised information across all sections to determine whether the individual should be referred for comprehensive diagnostic assessment. A standardised scoring framework was employed to enhance consistency whilst preserving clinical judgement. Each DSM-5 criterion was assigned a binary score: 0 (Insufficient Evidence) or 1 (Evidence Present).

A person was triaged as “Appropriate” for a full ADHD assessment if screening demonstrated evidence consistent with all assessed DSM-5 criteria (A, B, C, and D). This required the screening interview to identify observable ADHD symptoms (evidence for criterion A), reported childhood onset of symptoms prior to age 12 (evidence for criterion B), reported impairment across multiple life domains (evidence for criterion C), and reported clinically significant functional impact (evidence for criterion D).

Conversely, a triage outcome of “Not Appropriate” was assigned if screening failed to demonstrate evidence consistent with one single DSM-5 criterion. That is, if the screening interview did not reveal observable symptoms (criterion A), or did not identify childhood onset (criterion B), or did not document cross-situational impairment (criterion C), or did not establish functional impact (criterion D), the individual was excluded from comprehensive assessment.

At this stage of the pilot, criterion E (exclusion of other mental disorders) was treated differently. People were not excluded from comprehensive assessment based solely on the presence of alternative explanations for their difficulties, recognising that differential diagnosis and exclusion of competing conditions require the detailed evaluation possible during comprehensive assessment rather than brief screening.

The framework provided that evidence however weak for all four criteria (A, B, C, and D) indicated that referral for a full ADHD assessment was appropriate. Conversely, people where screening failed to demonstrate evidence for one or more criteria were triaged as “Not Appropriate”, as they were considered unlikely to meet full diagnostic criteria following a full diagnostic assessment. People not proceeding to an ADHD assessment received appropriate signposting to alternative support pathways and were not excluded from care.

To illustrate the decision-making process we present an example of a participant reporting childhood onset of symptoms (Criterion B met), showed observable inattentive and hyperactive behaviours during interview consistent with DSM-5 descriptions (Criterion A met), describing difficulties across both occupational and domestic settings (Criterion C met), and scoring above threshold on the functional impairment scale (Criterion D met) would be triaged as Appropriate regardless of Criterion E status. Conversely, a participant reporting onset of attentional difficulties only in the context of a recent depressive episode (Criterion B not met), with no observable symptoms during interview (Criterion A not met), would be triaged as Not Appropriate and offered signposting to appropriate mental health support.

#### 2.4.4 Documentation and record-keeping

All screening interviews were documented using the CASQ template as a prompt, with completed forms scanned into electronic patient records. The clinician recorded all quantitative ratings (observational symptom ratings, functional impairment scores, criterion scoring); free-text responses to open-ended questions; clinical reasoning regarding criterion fulfilment; referral decision and rationale. This documentation served both clinical and research purposes, enabling systematic review of decision-making processes and facilitating quality assurance activities.

#### 2.4.5 Diagnostic gold standard and blinding

To validate the triage decisions, all 48 participants were offered a comprehensive diagnostic assessment in accordance with the NICE guideline NG87. Comprehensive assessments were conducted according to established best-practice guidance for adult ADHD diagnosis as specified in the Adult ADHD Quality Assessment Scheme (AQAS) (93) and NICE guideline NG87 (97).

This secondary phase used the Diagnostic Interview for ADHD in Adults (DIVA-2), a semi-structured clinical interview designed to systematically evaluate DSM-5 criteria for ADHD across the lifespan, which systematically explores childhood and current symptomatology, developmental history, functional impairment in multiple life domains, and differential diagnosis considerations, and a complete psychiatric history. Assessments were conducted by experienced clinicians qualified in adult ADHD diagnosis, with typical assessment duration of over 120 minutes.

To ensure diagnostic rigour, participants were kept blind to the outcome of the initial screening decision during the second phase. Whilst efforts were made to mask the diagnostic assessors, absolute blinding could not be guaranteed as assessors had potential access to electronic patient records containing the screening documentation. Clinicians conducting comprehensive assessments had access to screening documentation within the electronic patient record and were therefore not masked to screening results. This pragmatic limitation reflects the real-world clinical setting where comprehensive assessment often builds upon existing clinical data and routine clinical practice wherein comprehensive assessment builds upon information gathered during screening. No formal safeguards beyond the use of structured diagnostic procedures (DIVA-2) and predefined diagnostic criteria (DSM-5) were implemented to mitigate this potential bias. However, the structured nature of the DIVA-2 and the requirement to apply DSM-5 criteria systematically provide inherent protection against unstructured confirmatory bias.

Diagnostic decisions were based on integrating the DIVA-2 findings, collateral information where available, review of developmental and psychiatric history, and clinical judgement regarding fulfilment of DSM-5 criteria and differential diagnosis. Diagnostic outcomes were classified into four categories: (1) ADHD - full diagnostic criteria met with confidence; (2) Trial - diagnostic uncertainty warranting therapeutic trial of medication to clarify diagnosis; (3) No ADHD - diagnostic criteria not met or symptoms better explained by alternative diagnosis; and (4) Incomplete - assessment not completed due to non-attendance or withdrawal from assessment process. For reporting purposes, the number of participants receiving each diagnostic classification was recorded separately to document the proportion of definitive versus trial-based diagnostic decisions.

Cases classified as “Trial” represented cases where diagnostic uncertainty persisted following comprehensive assessment, typically due to complex comorbidity, limited collateral information, or ambiguous developmental history. In such cases, response to ADHD medication can be used as additional diagnostic information, consistent with established clinical practice for off label use of treatment. However, for all subsequent statistical analyses including diagnostic accuracy metrics and comparative t-tests, “Trial” classifications were grouped with confirmed “ADHD” diagnoses, as both outcomes resulted in ADHD treatment initiation and represented clinical decisions that ADHD was sufficiently likely to warrant therapeutic intervention. This grouping errs on the side of caution for the study’s primary safety question, as counting an uncertain case as a true positive sets a higher bar for sensitivity than excluding it. This grouping is explicitly indicated in the Results section where both definitive ADHD diagnoses and Trial classifications are enumerated separately before being combined as “ADHD Diagnosed” for analytical purposes. A sensitivity analysis excluding the Trial case is also reported.

Cases where comorbid psychiatric conditions were identified alongside ADHD were classified as “ADHD” provided the comprehensive assessment concluded that ADHD criteria were met. Cases where symptoms were determined to be better explained by alternative psychiatric diagnoses (for example, attention difficulties secondary to depression or anxiety) were classified as “No ADHD”.

### 2.5 Statistical analysis and outcome measures

#### 2.5.1 Primary outcome: number needed to harm

The primary outcome measure was the Number Needed to Harm (NNH), representing the number of people triaged before a single individual with ADHD is incorrectly excluded (a false-negative result). NNH was calculated as the inverse of absolute risk of harm, where harm was operationalized as false-negative triage decision (individual triaged “Not Appropriate” who subsequently received ADHD diagnosis). Where no false negatives occurred, NNH was reported as infinite. NNH was selected as the primary outcome because it directly operationalizes the clinical question of greatest concern in screening pathway design: how many people must undergo this process before one person with genuine ADHD is incorrectly excluded? This framing has direct clinical meaning that sensitivity alone does not convey as intuitively. In the context of small samples, an infinite NNH reflects the absence of observed false negatives rather than a guarantee of zero false-negative risk in larger populations, and should be interpreted accordingly.

#### 2.5.2 Secondary outcomes and descriptive statistics

Secondary outcomes included the calculation of sensitivity, specificity, and predictive values (PPV and NPV) with 95% Confidence Intervals (CI) estimated by the Wilson score method. The proportion of referrals safely screened out (those triaged as “Not Appropriate” who did not receive ADHD diagnoses) was calculated to assess operational efficiency.

Descriptive analyses were conducted to characterise the sample and summarise screening scores and diagnostic outcomes. Gender distribution was reported as a participant characteristic. For screening assessment data, means, standard deviations, and ranges were calculated for Total Inattention Score, Total Hyperactivity/Impulsivity Score, Total Impairment Score, and Overall Impairment Level.

Triage decisions were analysed by calculating the frequency and percentage of cases classified as “Appropriate” or “Not Appropriate” for further assessment. Diagnostic outcomes were summarised by reporting frequencies and percentages for final diagnostic classifications (No ADHD, ADHD, Trial, or Incomplete). The overall ADHD diagnostic rate was calculated as the proportion of completed assessments resulting in an ADHD diagnosis (including both confirmed ADHD and Trial classifications), expressed as a percentage.

#### 2.5.3 Statistical assessment of inter-rater variability

To ensure the reliability of the triage process across different clinical assessors, an assessment of inter-rater variability was conducted. The cohort was stratified by the specific screening clinician and the resulting triage outcome. A Pearson Chi-square test of independence was utilised to determine whether triage decisions were significantly associated with the individual clinician, which would indicate a lack of procedural standardisation. The effect size was further quantified using Cramer’s V to evaluate the strength of association between these variables.

#### 2.5.4 Handling of missing data

Of the 49 participants who underwent screening, 48 completed comprehensive diagnostic assessment. One participant (2.0%) did not complete the comprehensive assessment due to relocation prior to completion. This case was classified as “Incomplete” and excluded from diagnostic accuracy analyses.

#### 2.5.5 Diagnostic accuracy analysis

A 2×2 contingency table was constructed crossing triage decision (Appropriate vs Not Appropriate) with diagnostic outcome (ADHD Diagnosed vs No ADHD). The single case with incomplete assessment was excluded from accuracy analysis. “ADHD Diagnosed” included cases classified as “ADHD” or “Trial”, reflecting the clinical decision to initiate ADHD treatment. Cases where comorbid psychiatric conditions were identified alongside ADHD were classified as “ADHD Diagnosed” provided the comprehensive assessment concluded that ADHD criteria were met. Cases where symptoms were determined to be better explained by alternative psychiatric diagnoses (for example, attention difficulties secondary to depression or anxiety) were classified as “No ADHD”.

The following accuracy metrics were calculated with 95% confidence intervals using the Wilson score method:

- Sensitivity: True Positives/(True Positives + False Negatives)
- Specificity: True Negatives/(True Negatives + False Positives)
- Positive Predictive Value (PPV): True Positives/(True Positives + False Positives)
- Negative Predictive Value (NPV): True Negatives/(True Negatives + False Negatives)

#### 2.5.6 Exploratory analyses

To assess whether positive predictive value could be increased by applying more stringent triage thresholds or whether screening scores could differentiate between diagnostic outcomes, exploratory analyses were undertaken. These analyses addressed the clinical question of whether quantitative screening scores could be used to refine triage decisions and improve the modest positive predictive value inherent in safety-focussed screening pathways.

First, screening scores (Total Inattention Score, Total Hyperactivity/Impulsivity Score, Total Impairment Score, and Overall Impairment Level) were compared across the entire analytical sample (N = 48) between participants who received ADHD diagnoses and those who did not, using independent samples t-tests. This analysis examined whether screening scores differed between diagnostic outcome groups regardless of triage decision, providing evidence on the overall discriminative capacity of the screening instrument.

Second, to determine whether screening scores could improve triage accuracy within the “Appropriate” group, participants triaged as “Appropriate” were further classified as True Positives (those subsequently diagnosed with ADHD) or False Positives (those not diagnosed with ADHD). This classification was based solely on the final comprehensive diagnostic outcome, permitting retrospective examination of whether the two groups differed at the screening stage. Independent samples t-tests compared screening scores between these two groups on each of the four quantitative screening measures (Total Inattention Score, Total Hyperactivity/Impulsivity Score, Total Impairment Score, and Overall Impairment Level). This sub-analysis examined whether score magnitude within the “Appropriate” triage category was associated with final diagnostic outcome, potentially identifying score thresholds that could reduce false-positive rates without compromising sensitivity. The rationale for this focussed analysis is that the “Appropriate” group represents the critical decision point where additional discrimination would be most valuable as if True Positives consistently demonstrated higher screening scores than False Positives, quantitative cut-offs could be applied within the Appropriate category to improve positive predictive value whilst maintaining the safety of the initial inclusive threshold.

Third, positive predictive value was recalculated under a hypothetical lenient triage threshold requiring evidence for any single DSM-5 criterion (A, B, C, or D) at screening, rather than the current requirement for evidence consistent with all four criteria. This analysis assessed whether relaxing the threshold to permit comprehensive assessment based on meeting any single criterion would improve sensitivity, whilst considering the potential trade-off in positive predictive value and operational efficiency.

All statistical procedures were performed using IBM SPSS Statistics version 30.0 (IBM Corporation, Armonk, NY, USA).

#### 2.5.7 Data management and quality assurance

All CASQ forms were completed during clinical assessments and scanned into patient records. Data extraction was conducted by clinicians employed within the service using a standardised template capturing demographic information, observational ratings, total screening scores, triage decisions with supporting rationale, comprehensive assessment outcomes, and diagnostic decisions. Quality assurance included regular review of completed forms to ensure consistency in rating scale application and criterion scoring.

### 2.6 Ethical considerations and data governance

The South West Yorkshire Partnership NHS Foundation Trust Research and Development Department classified this project as a service improvement activity; consequently, formal ethical approval was waived. The Caldicott Guardian at SWYPFT authorised access to patient data in strict adherence to the Caldicott Principles of data governance. Individual patient consent was not applicable given the classification as service improvement.

The study design prioritised patient safety by ensuring universal access to a full diagnostic assessment during the validation phase. All cases received the full diagnostic assessment regardless of triage decision, thereby enabling empirical verification of triage safety without exposing people to risk of inappropriate exclusion. This universal assessment approach represented best practice during the evaluation phase, though routine clinical implementation would have involved not having an assessment based on triage decisions.

## 3 Results

### 3.1 Participant flow and characteristics

Between January 2024 and February 2025, a consecutive sample of 49 adults was recruited for assessment. One person was excluded from the final analysis because they moved away from the catchment area prior to completion of the diagnostic process, leaving their clinical status undetermined. The remaining 48 participants formed the primary analytical cohort. All 48 participants who completed the study received the same comprehensive NICE-compliant diagnostic assessment regardless of their initial triage classification, thereby eliminating verification bias.

The sample was predominantly female (n = 34, 70.8%). All participants were undergoing their first specialist ADHD assessment, and none had a previous ADHD diagnosis. Following the full diagnostic process using the DIVA-2 and the full diagnostic assessment, a diagnosis of ADHD was confirmed in 12.5% (n = 6) of participants. This included five participants receiving a definitive ADHD diagnosis and one participant classified as “Trial,” wherein diagnostic uncertainty warranted a therapeutic trial of medication to clarify diagnosis. Most of the cohort (n = 42, 87.5%) did not meet full DSM-5 criteria for ADHD. Of the 48 completed assessments, 29 (60.4%) were initially triaged as “Appropriate” for further assessment, whereas 19 (39.6%) were triaged as “Not Appropriate” at the screening stage.

Amongst the 42 participants who did not receive an ADHD diagnosis, the full ADHD assessment identified a range of alternative clinical presentations. The most frequently identified primary clinical impressions included anxiety disorders, depressive disorders, personality-related difficulties, adjustment difficulties and situational stressors, and attentional difficulties attributed to other psychiatric conditions. This distribution is consistent with the broader literature demonstrating that in adults presenting for first-time ADHD assessment, the majority of ADHD-like symptoms are attributable to other conditions, particularly mood and anxiety disorders (101). These findings reinforce the importance of differential diagnosis within any screening pathway and contextualise the high false-positive rate observed in this study.

### 3.2 Screening assessment findings

The CASQ screening approach permitted a detailed assessment of symptom burden and functional deficits across all participants (**Table 1**). The mean Total Inattention Score for the entire sample was 2.79 (SD = 3.49, range 0-11), and the mean Total Hyperactivity/Impulsivity Score was 3.67 (SD = 3.66, range 0-12). Functional impact, a core component of the triage logic, produced a mean Total Impairment Score of 6.08 (SD = 4.34, range 0-15) and an Overall Impairment Level of 1.19 (SD = 0.94, range 0-3).

**Table 1:** CASQ screening scores and functional impairment ratings (N = 48).

Measure	Mean	SD	Range
Total Inattention Score	2.79	3.49	0–11
Total Hyperactivity/Impulsivity Score	3.67	3.66	0–12
Total Impairment Score	6.08	4.34	0–15
Overall Impairment Level	1.19	0.94	0–3

When stratified by gold-standard diagnostic outcome, the ADHD group had higher mean scores across all recorded domains, though these differences did not reach statistical significance given the small sample size of the ADHD group (**Table 2**). Those with a confirmed diagnosis had a mean Inattention Score of 3.33 (SD = 4.23) and a mean Hyperactivity/Impulsivity Score of 4.83 (SD = 3.81), compared with 2.71 (SD = 3.43) and 3.50 (SD = 3.65), respectively, in the non-ADHD group. These scores highlight the inclusive nature of the triage pathway, as people with relatively low symptom scores were still permitted to proceed to full assessment to ensure maximal safety.

**Table 2:** Comparison of CASQ screening scores by final diagnostic outcome.

Measure	ADHD diagnosed (n=6) mean (SD)	No ADHD (n=42) mean (SD)	t	p
Total Inattention Score	3.33 (4.23)	2.71 (3.43)	.402	0.345
Total Hyperactivity/Impulsivity Score	4.83 (3.81)	3.50 (3.65)	.833	0.205
Total Impairment Score	7.33 (5.28)	5.90 (4.35)	.734	0.233
Overall Impairment Level	1.50 (1.05)	1.14 (0.93)	.871	0.194

### 3.3 Primary outcome: pathway safety and number needed to harm

The primary objective of this study was to determine the safety of the criterion-based triage, defined by the Number Needed to Harm (NNH). This represents the number of people who must undergo the screening process before one individual with genuine ADHD is incorrectly excluded (a false-negative result).

Across all 48 completed assessments, no case triaged as “Not Appropriate” was subsequently found to meet diagnostic criteria for ADHD during the blinded gold-standard assessment. All six people who received a final diagnosis had been correctly identified as “Appropriate” during the initial triage (**Table 3**). Accordingly, the NNH was infinite. This finding indicates that the triage process, despite being conducted in a real-world clinical setting by Physician Assistants, did not result in any observed harm through the inappropriate exclusion of valid ADHD cases in this cohort.

**Table 3:** Relationship between triage decisions and final diagnostic outcomes.

Triage decision	ADHD diagnosed	No ADHD	Total
Appropriate	6 (True Positive)	23 (False Positive)	29
Not Appropriate	0 (False Negative)	19 (True Negative)	19
Total	6	42	48

When the Trial classification was excluded from the ADHD-positive group (retaining only the five confirmed diagnoses), sensitivity remained 100.0% (95% CI: 56.6%–100.0%), as the Trial case had also been correctly triaged as Appropriate. The remaining diagnostic accuracy metrics were not materially altered. This sensitivity analysis confirms that the primary findings are not dependent on the classification of the single diagnostically uncertain case.

### 3.4 Secondary outcomes: diagnostic accuracy and operational performance

The diagnostic accuracy of the screening pathway was calculated against the clinical gold standard (**Table 4**). The triage demonstrated a sensitivity of 100.0% (95% CI: 61.0%–100.0%), confirming that the pathway was successful in capturing all people who required a diagnosis. Specificity was more modest at 45.2% (95% CI: 31.2%–60.1%), which reflects the inclusive threshold where a substantial number of non-ADHD cases were offered a comprehensive assessment to prioritise safety.

**Table 4:** Diagnostic accuracy metrics for criterion-based triage.

Sensitivity	100.0%	61.0% – 100.0%
Specificity	45.2%	31.2% – 60.1%
Positive Predictive Value	20.7%	9.8% – 38.4%
Negative Predictive Value	100.0%	83.2% – 100.0%
Number Needed to Harm	∞	–
Proportion safely screened out	39.6%	27.0% – 53.7%

The positive predictive value (PPV) was 20.7% (95% CI: 9.8%–38.4%), indicating that approximately one in five people triaged as “Appropriate” was subsequently diagnosed with ADHD. The negative predictive value (NPV) was 100.0% (95% CI: 83.2%–100.0%), confirming the reliability of the “Not Appropriate” triage decision. In terms of operational efficiency, the pathway permitted 39.6% (n = 19) of referrals to be safely screened out without the need for a comprehensive diagnostic assessment, thereby potentially reducing the burden on specialist clinical resources.

### 3.5 Inter-rater variability and procedural consistency

A comparative analysis examined whether triage outcomes varied across the four clinicians who administered the CASQ. The descriptive results showed that the proportion of cases judged as appropriate ranged from 50.0% to 87.5% across clinicians. A Pearson Chi−square test found no statistically significant association between clinician and triage outcome (*χ²* = 3.024, df = 3, *p* = 0.38). Cramer’s V was 0.251, indicating only a moderate, non−significant association. Overall, the results suggest that triage decisions were consistent across raters, reinforcing the reliability of the CASQ when applied by trained Physician Assistants.

### 3.6 Exploratory analyses: positive predictive value

#### 3.6.1 Score comparison between true positives and false positives

Amongst the 29 participants triaged as “Appropriate”, 6 (20.7%) were subsequently diagnosed with ADHD (true positives) and 23 (79.3%) were not (false positives). Independent samples t-tests compared mean screening scores between these groups to determine whether score magnitude could refine triage accuracy (**Table 5**). None of these differences reached statistical significance, likely due to the small sample size.

**Table 5:** Comparison of screening scores between true positives and false positives amongst those triaged “appropriate”.

Measure	True positives (n=6) mean (SD)	False positives (n=23) mean (SD)	Mean difference	t	p
Total Inattention Score	3.33 (4.23)	4.09 (3.71)	-0.76	-.431	0.335
Total Hyperactivity/Impulsivity Score	4.83 (3.82)	4.52 (3.34)	+0.31	.198	0.422
Total Impairment Score	7.33 (5.28)	7.35 (4.68)	-0.02	-.007	0.497
Overall Impairment Level	1.50 (1.05)	1.43 (0.99)	+0.07	.142	0.444

The absence of statistically significant differences suggests that, within the group triaged as “Appropriate”, screening scores alone do not reliably distinguish between true and false positives.

### 3.7 Criterion threshold analysis

A *post-hoc* analysis examined whether relaxing the triage threshold to permit referral based on meeting any DSM-5 criterion (A, B, C or D), rather than requiring all criteria (A, B, C and D) would improve sensitivity whilst maintaining acceptable operational efficiency. Under the current strict threshold requiring fulfilment of all four assessed criteria (A, B, C and D), 29 participants (60.4%) were triaged as “Appropriate” for comprehensive assessment. Of these 29, six received an ADHD diagnosis, yielding a positive predictive value of 20.7%.

Under a hypothetical lenient threshold wherein meeting any single criterion would be sufficient for triage as “Appropriate”, 43 participants (89.6%) would have been referred for comprehensive assessment. All six people who received ADHD diagnoses met at least one criterion at screening, indicating that the lenient threshold would have maintained perfect sensitivity (100.0%). However, the lenient threshold would have captured an additional 14 non-ADHD cases beyond those already triaged as “Appropriate” under the strict threshold, reducing positive predictive value from 20.7% to 14.0% whilst substantially diminishing operational efficiency from 39.6% safely screened out to only 10.4%.

The strict threshold requiring fulfilment of all assessed criteria therefore demonstrated superior performance to the lenient alternative. Whilst both approaches achieved identical sensitivity (100.0%), maintaining perfect detection of all ADHD cases, the strict threshold offered substantially better positive predictive value (20.7% versus 14.0%) and markedly improved operational efficiency (screening out 39.6% versus 10.4% of referrals). The stricter approach therefore achieves the important safety requirement of zero false negatives whilst maximising resource efficiency and minimising the burden on specialist assessment services.

The distribution of exclusion patterns provides insight into the triage mechanism. Amongst the 19 people triaged as “Not Appropriate”, 68% failed to demonstrate evidence for multiple DSM-5 criteria, whilst 32% met evidence thresholds for two or three criteria but failed on at least one. A proportion of those who were not offered a full assessment (10.5%) had clear evidence of childhood onset and cross−situational impairment but showed no observable ADHD symptoms during screening (criterion A), underscoring the importance of direct clinical observation alongside self−report.

## 4 Discussion

This study provides the first formal validation of a criterion-based screening and triage pathway for adult ADHD implemented in a real-world secondary care setting. This mirrors the criterion based approach we had used for Autism (28). The primary finding show that such pathways can achieve complete safety, with zero false-negative results (NNH = ∞, sensitivity 100%), whilst meaningfully improving operational efficiency by safely screening out 39.6% of referrals. These results address an important evidence gap in adult ADHD services and offer empirical support for structured screening approaches that move beyond traditional self-report questionnaires to systematic clinical assessment of DSM-5 diagnostic criteria.

The absence of any false negatives across 48 consecutive assessments indicates that criterion based screening, when delivered by appropriately trained non specialist clinicians, can reliably identify adults who require a full diagnostic assessment for ADHD. This is an important finding, given the ethical need to prioritise sensitivity over specificity in healthcare screening, arguably more in neurodevelopmental conditions, where missed diagnoses can sustain functional impairment, increase risks of adverse outcomes such as occupational difficulties and comorbid psychiatric conditions, and delay access to effective interventions. The 100% sensitivity demonstrated here also compares favourably with self report screening tools, whose sensitivity varies widely across clinical settings and in populations with high levels of psychiatric comorbidity (47, 58).

The criterion based approach used in this study helps overcome an important limitation of self report screening tools, which focus solely on symptom checklists and are therefore vulnerable to the substantial overlap between ADHD symptoms and those of other psychiatric conditions. By requiring systematic examination of presence of ADHD symptoms (criterion A), age of onset (criterion B), cross-situational impairment (criterion C), functional impact (criterion D), and differential diagnosis (criterion E), the CASQ enables clinicians to distinguish neurodevelopmental ADHD from attention difficulties secondary to mood disorders, anxiety, trauma, or situational stressors. This discriminative capacity likely contributed to the pathway’s excellent sensitivity, as the structured clinical interview format permitted real-time clarification of ambiguous responses and considering the patterns of symptoms over time that distinguish lifelong neurodevelopmental difficulties from recent-onset problems. Direct comparison with the ASRS was not undertaken in the present study because the two approaches occupy different positions in the clinical pathway: one is a self-report symptom checklist, the other is a clinician-administered criterion-based assessment. A head-to-head comparison within the same cohort would be methodologically appropriate and is recommended as a priority for future research.

The modest specificity (45.2%, 95% CI: 31.2%–60.1%) and positive predictive value (20.7%, 95% CI: 9.8%–38.4%) observed in this study reflect the intentional calibration of the triage threshold to ensure safety whilst maintaining operational efficiency. The decision rule, wherein people were not offered comprehensive assessment if screening failed to demonstrate evidence consistent with any single assessed DSM-5 criterion (A, B, C, or D), was designed to balance the safety requirement of zero false-negatives against the practical necessity of screening out a meaningful proportion of referrals in a capacity-constrained service. This strict threshold achieved perfect sensitivity, capturing all six ADHD cases, whilst appropriately redirecting 39.6% of referrals away a full diagnostic assessment.

The positive predictive value of 20.7% indicates that approximately one in five people triaged as “Appropriate” received an ADHD diagnosis, a figure that compares favourably with the 10%-30% positive predictive values reported for self-report screening instruments at comparable base rates (67, 68). Positive predictive value is inherently dependent on the base rate of ADHD within the assessed population. Services with higher diagnostic yield may observe higher PPV, whilst those with lower base rates may observe lower PPV. The present estimates should be interpreted in the context of the 12.5% diagnostic rate observed in this consecutively referred sample. Importantly, the perfect excellent predictive value (100.0%, 95% CI: 83.2%–100.0%) confirms that the “Not Appropriate” triage decision reliably excluded ADHD, providing confidence that the 39.6% of referrals screened out can be safely managed through alternative pathways without risk of denying appropriate care.

*Post-hoc* analysis demonstrated that relaxing the threshold from excluding people based on absence of clinical evidence for any DSM-5 criterion (A, B, C or D) to requiring absence of evidence for all criteria (A, B, C and D) would have maintained perfect sensitivity but at substantial cost to operational efficiency. The lenient threshold would have reduced the proportion of referrals safely screened out from 39.6% to only 10.4%, whilst simultaneously decreasing positive predictive value from 20.7% to 14.0%. The strict threshold requiring evidence for all assessed criteria therefore achieves optimal balance, capturing all genuine ADHD cases whilst appropriately redirecting two-fifths of referrals away from a full assessment. This trade-off is consistent with established principles for screening pathway design in contexts where the consequences of missed diagnoses are serious and comprehensive assessment represents a relatively low-risk intervention.

The pathway’s capacity to safely screen out 39.6% of referrals is a meaningful operational benefit for services managing high assessment demand. Assessment of the Irish pathway from general practitioners to specialist ADHD services found demand exceeding capacity by factors of three to four, indicating that current referral patterns require refinement for efficiency (29). Similar capacity challenges have been documented internationally, with calls for differentiated pathways based on case complexity and development of shared care models to manage straightforward cases in primary care settings (30, 98).

In this study, screening interviews required 60 minutes per case (45 minutes face-to-face, 15 minutes administrative synthesis), whilst the full diagnostic assessments typically required well over 120 minutes. For the 19 cases screened out, the pathway therefore saved many hours of specialist clinician time that could be redirected towards cases who are more likely to be diagnosed. This resource reallocation addresses sustainability concerns in services where waiting times extend to months or years, potentially reducing diagnostic delay for people with genuine ADHD whilst ensuring those screened out receive appropriate signposting to alternative support.

More importantly, the operational benefits observed in this study were achieved without compromise to patient safety. The perfect negative predictive value confirms that people triaged as “Not Appropriate” genuinely did not meet ADHD diagnostic criteria, distinguishing efficient screening from inappropriate gatekeeping. This distinction is important in contexts where services face pressures to restrict access to specialist assessment, as screening pathways must be validated to ensure they facilitate appropriate care rather than merely reducing assessment volumes.

Looking at the wider context of screening and triage decisions in neurodevelopmental disorders, this study directly addresses the four evidence gaps identified in the introduction. These were whether clinicians can reliably apply DSM 5 criteria in time limited contexts, what threshold should trigger progression to full assessment, how criterion based pathways compare with self report tools in diagnostic accuracy across diverse populations, and what resource implications arise when implementing such pathways within different healthcare systems.

The issue of noncredible responding and symptom validity was something we did consider. Adult ADHD assessments frequently occur in contexts where secondary gain may be relevant, and clinicians are generally poor at detecting symptom exaggeration without structured validity indicators (100). The criterion-based approach employed in this study offers inherent safeguards that are absent from self-report instruments. The 45-minute clinical interview permits observation of behavioural congruence, probing of inconsistencies, and assessment of the quality and specificity of symptom accounts. Additionally, the requirement to demonstrate childhood onset, cross-situational impairment, and exclusion of alternative diagnoses provides multiple validity checkpoints that a symptom checklist cannot replicate. Nevertheless, the CASQ does not incorporate formal embedded validity indicators, and future iterations should consider integrating structured credibility checks, particularly for deployment in settings where secondary gain is more prominent.

It must be emphasised, however, that the diagnostic accuracy reported in this study is contingent on faithful adherence to the interview protocol as described. The CASQ’s safety profile is a property of the pathway as implemented under the conditions of this validation such as structured training, supervised practice, calibration exercises, and a specialist service culture prioritising rigorous differential diagnosis and cannot be assumed to transfer automatically to settings where these conditions are not replicated. Implementation fidelity is a well-recognised determinant of intervention effectiveness, and protocol degradation is common when structured clinical procedures are translated into routine practice under time pressure. In the specific context of adult ADHD assessment, the risk of fidelity drift is compounded by the current referral landscape, in which many adults present with pre-formed symptom narratives shaped by social media exposure and online ADHD communities. Research demonstrates that exposure to confirmatory information about ADHD can lead individuals to genuinely reframe their developmental history, endorse symptoms they might not otherwise have reported, and attribute diverse functional difficulties to a single neurodevelopmental explanation (12, 13). A screening interview that does not actively probe beneath this surface narrative by critically examining developmental timing, exploring alternative explanations, and evaluating whether reported impairment is consistent with objective indicators will inevitably capture self-reported accounts as evidence of ADHD, inflating false-positive rates. Clinicians are known to perform at near-chance levels in detecting symptom exaggeration without structured validity indicators, even when administering standardised instruments (100). Services adopting the CASQ should therefore ensure that the full 45-minute interview duration is preserved, that clinicians are trained to probe beyond self-reported symptom narratives, that developmental history is critically examined rather than accepted at face value, and that structured quality assurance mechanisms including ongoing calibration, supervised practice, and periodic audit of screening decisions against diagnostic outcomes are embedded within the implementation framework. Without these safeguards, the false-positive rate should be expected to increase, and the diagnostic accuracy reported in this study should not be assumed to generalise.

### 4.1 Reliability of non-specialist clinician assessment

The finding that Physician Assistants with specialist training but without psychiatrist-level expertise could reliably apply DSM-5 criteria within time-constrained screening contexts demonstrates the feasibility of task-shifting models for ADHD assessment. Primary care providers frequently report insufficient confidence in managing complex ADHD presentations (98), raising questions about capacity for accurate criterion-based screening. The present study suggests that with appropriate training (4 hours didactic instruction, 10 supervised practice assessments), non-specialist clinicians can conduct safe and effective screening interviews.

This finding has important implications for workforce models in ADHD services. Restricting adult ADHD assessment exclusively to tertiary specialist services limits scalability and contributes to diagnostic bottlenecks. By shifting screening functions to appropriately trained non-specialists whilst reserving the full assessments for specialist clinicians represents a pragmatic approach to capacity constraints, provided such models are formally validated to ensure safety. The present study suggests that with appropriate training (4 hours didactic instruction, 10 supervised practice assessments, and calibration exercises involving joint scoring and consensus discussions), non-specialist clinicians can conduct safe and effective screening interviews.

### 4.2 Appropriate thresholds for comprehensive assessment

The inclusive threshold employed in this study requiring evidence for DSM-5 criterion A, criterion B, criterion C and criterion D, whilst deferring exclusion of alternative explanations (criterion E) achieved excellent safety but moderate specificity. This approach ensured that all people who went on to receive an ADHD diagnosis were correctly identified.

*Post-hoc* analyses demonstrated that relaxing the threshold further, such that individuals would be deemed “Appropriate” for full ADHD assessment if they met any of the DSM-5 criteria, would not have improved case detection. Although such a lenient approach would have maintained excellent sensitivity, it would have substantially reduced operational efficiency, markedly decreasing the proportion of referrals deemed “Not Appropriate” and lowering positive predictive value.

These findings indicate that the threshold used in this study achieved an appropriate balance between sensitivity and specificity. By requiring evidence of criterion A, criterion B, criterion C and criterion D, this threshold avoided over-inclusion associated with highly permissive thresholds. Importantly, this balance was achieved without compromising safety, as evidenced by the absence of false-negative outcomes.

### 4.3 Comparative performance against self-report screening

Whilst this study did not directly compare criterion-based screening against self-report instruments within the same cohort, the observed sensitivity (100%) and positive predictive value (20.7%) suggest favourable performance relative to published data on the ASRS and similar tools. The ASRS has high sensitivity in selected populations, but specificity reduces considerably in clinical samples with psychiatric comorbidity, precisely the contexts where criterion-based screening offers theoretical advantages through considering systematically differential diagnosis. The CASQ’s structured assessment of DSM-5 criterion E (exclusion of other mental disorders) represents a particular strength relative to self-report questionnaires, which cannot systematically evaluate whether attention difficulties are better explained by alternative diagnoses. This capability is important in adult mental health settings where diagnostic complexity is the norm rather than exception, and where attention difficulties may reflect mood disorders, anxiety, trauma, substance use, or situational stressors rather than neurodevelopmental ADHD.

### 4.4 Resource implications

The 60-minute screening duration represents the primary resource requirement of criterion-based pathways, substantially exceeding the minimal time required for self-report questionnaire completion (typically 5–10 minutes). However, this comparison oversimplifies resource considerations, as questionnaires generate positive screens requiring subsequent clinical review and triage decisions. In systems where positive ASRS screens automatically proceed to the full diagnostic assessment without intermediate triage, the modest positive predictive values (10-30%) result in substantial specialist time devoted to assessing people unlikely to meet diagnostic criteria.

The criterion-based pathway examined in this study consolidates initial screening and triage into a single 60-minute session conducted by non-specialist clinicians, avoiding the need for subsequent specialist triage of questionnaire-positive cases. For people screened out, the pathway saves specialist time relative to the full diagnostic assessment. In addition, for people proceeding to this, the screening documentation provides useful contextual information that may enhance efficiency of the assessment. The net resource implications therefore depend on the proportion screened out and the relative costs of non-specialist versus specialist clinician time. It was not within the scope of this practice to calculate the full resource immolations. A comparative economic evaluation is required to formally establish cost-effectiveness, incorporating both direct costs (clinician time, training, infrastructure) and indirect costs (diagnostic delay, treatment delay, opportunity costs of missed diagnoses). This will be the purpose of a separate publication, and the present study provides important diagnostic accuracy data to inform such economic modelling, demonstrating that criterion-based pathways can achieve high safety and meaningful operational efficiency under real-world conditions.

### 4.5 Limitations

There are several limitations with this study and how we interpret its findings. First, the sample size was modest (n = 48), determined by service capacity rather than formal power calculations. Whilst adequate to demonstrate zero false negatives in this cohort, the small number of ADHD cases (n = 6) limits precision of sensitivity estimates, reflected in the wide confidence interval (61.0%-100.0%). The upper bound confirms 100% sensitivity, but the lower bound indicates uncertainty about performance in larger samples. Applying a heuristic rule of three, observing zero false negatives amongst six ADHD diagnoses yields an upper 95% confidence bound for the true false-negative rate of approximately 50%, indicating that the observed zero false-negative rate, whilst encouraging, does not exclude the possibility of clinically meaningful miss rates in larger samples. The present findings should therefore be regarded as preliminary evidence of safety requiring confirmation in adequately powered replication studies. A single missed case in a larger sample would reduce sensitivity considerably, underscoring the statistical fragility of this estimate. Replication in larger cohorts would be required to establish stable sensitivity estimates and determine whether perfect sensitivity represents a reproducible characteristic of criterion-based screening or a fortuitous outcome specific to this sample.

Second, clinicians conducting the full diagnostic assessments were not fully masked to screening results, as they had potential access to electronic patient records containing screening documentation. Whilst participants were kept blind to triage decisions, the lack of complete assessor blinding could theoretically introduce bias if comprehensive diagnostic decisions were influenced by knowledge of screening outcomes. The use of structured diagnostic procedures (DIVA-2) and predefined diagnostic criteria (DSM-5) provides some inherent protection against unstructured confirmatory bias. However, the direction of such bias would likely be towards concordance between screening and the full assessment, potentially inflating apparent triage accuracy. The fact that 23 people triaged as “Appropriate” did not receive ADHD diagnoses despite this potential bias suggests that the clinicians conducting the assessments exercised independent clinical judgement rather than merely confirming screening decisions.

Third, following the gold−standard full diagnostic assessment, there was one case in which a definitive diagnostic conclusion could not be reached, resulting in its classification as a “trial” case alongside confirmed ADHD diagnoses. This approach is defensible, as both classifications lead to initiation of ADHD treatment, and a sensitivity analysis excluding this case demonstrated no material change in diagnostic accuracy results. However, it introduces a small degree of heterogeneity within the ADHD group.

Fourth, the study was conducted in a single secondary care service with a specific referral population and service configuration. Generalizability to primary care settings, community mental health teams, or populations with different demographic characteristics, comorbidity patterns, or levels of diagnostic complexity would requires empirical verification. The high proportion of female participants (72.9%) also may not reflect a typical adult ADHD service referral pattern considering the expected prevalence favouring males (99) which may limit generalizability to male-predominant samples or settings with different gender distributions.

Fifth, the study is subject to criterion contamination arising from common method variance. Both the index test (CASQ) and the diagnostic reference standard (DIVA-2) are clinician-administered interviews that assess DSM-5 symptomatology and functional impairment. Because the predictor and the criterion share the same measurement method, concordance between them may be partly attributable to shared method variance rather than the genuine diagnostic accuracy of the screening pathway. A person who presents with a convincing clinical narrative in one interview format is likely to do so in another, potentially inflating the apparent sensitivity of the CASQ. This limitation is inherent in all criterion-related validation in behavioural health where no objective biomarker exists, as any screening pathway for ADHD must ultimately be validated against a clinical interview. Nevertheless, two features of the present data provide partial reassurance. First, the substantial discordance between the CASQ and the DIVA-2 amongst the Appropriate group (23 of 29 cases triaged as Appropriate were not diagnosed) demonstrates that the DIVA-2 exercised independent diagnostic judgement rather than simply confirming CASQ decisions. Second, the CASQ (a 45-minute screening encounter) and the DIVA-2 (a 120-minute diagnostic interview incorporating collateral information and comprehensive developmental history) differ materially in depth, duration, and informational scope, partially mitigating the shared method concern. Nonetheless, the true diagnostic accuracy of the CASQ may be lower than the estimates reported here, and future validation work should consider incorporating assessment methods that differ in format from the index test, such as informant ratings, objective cognitive measures, or ecological momentary assessment, to provide convergent evidence that is less susceptible to common method variance.

Sixth, the CASQ does not incorporate formal embedded validity indicators for the detection of noncredible responding or symptom exaggeration. Although the structured interview format permits examination of internal consistency, developmental plausibility, symptom persistence, and alternative explanations, these safeguards are not equivalent to formal performance or symptom validity procedures, nor should clinicians be assumed to detect exaggerated responding accurately through clinical impression alone. In addition, the performance observed in this study was obtained under a relatively rigorous specialist-service protocol involving trained clinician administration, protected interview time, and structured post-interview synthesis. The pathway should therefore not be assumed to retain the same operating characteristics if implemented in more abbreviated or less controlled ways. In particular, use of the CASQ as a patient-completed form, use without adequate probing of chronology and differential diagnosis, or use without collateral or documentary corroboration where available will increase false-positive triage outcomes.

### 4.6 Future directions

Several research priorities emerge from this validation study. First, multi-site replication with larger samples is essential to confirm the generalizability of findings and establish stable estimates of diagnostic accuracy parameters. Particular priority should be given to primary care settings, where criterion-based screening could support diagnostic task-shifting but where clinician confidence in ADHD assessment is often limited (99). Validation in primary care would determine whether brief structured screening interviews can be conducted safely by general practitioners or primary care mental health practitioners with appropriate training. Evaluation in settings with higher ADHD base rates would also clarify the performance of the pathway under different prevalence conditions.

Second, direct comparative effectiveness research is needed to establish the incremental validity of criterion-based screening over self-report questionnaires. Sequential administration of the ASRS and CASQ within the same cohort, with both pathways masked to comprehensive diagnostic outcomes, would clarify whether the additional time and resource requirements of structured clinical interviews produce meaningful improvements in diagnostic accuracy or whether questionnaire-based approaches achieve comparable performance with lower resource demands.

Third, formal psychometric validation of the observational rating subscales (Inattention and Hyperactivity-Impulsivity) is required, including inter-rater reliability coefficients derived from independent double-rating of clinical interviews. This work is currently in preparation and will be reported separately.

Fourth, implementation science research is needed to understand factors enabling or hindering adoption of criterion-based screening in diverse service contexts. Qualitative research with clinicians, service managers, and people using services could identify barriers to implementation (training requirements, workflow integration, acceptability) and facilitators that support successful adoption. Process evaluation of implemented pathways should examine fidelity to screening protocols, clinician adherence to decision rules, and patient experience of multi-stage assessment processes.

Fifth, longitudinal research should examine outcomes for people triaged as “Not Appropriate”, tracking subsequent mental health service utilisation, diagnostic outcomes, and functional trajectories. Such research would verify that screening decisions do not merely delay inevitable ADHD diagnoses but genuinely distinguish people requiring neurodevelopmental assessment from those whose difficulties reflect alternative explanations. Follow-up studies could also examine re-referral rates and diagnostic stability over time.

Sixth, future iterations of the CASQ should consider integrating structured validity indicators for the detection of noncredible responding, particularly for deployment in contexts where secondary gain may be more salient, such as forensic settings, disability assessments, or academic accommodation requests.

Finally, integration of criterion-based screening frameworks with emerging technological innovations represents an important frontier. Development and validation of AI-assisted triage systems that operationalize DSM-5 criteria rather than merely automating symptom checklists could enhance efficiency whilst preserving diagnostic rigour. Natural language processing applied to screening interview transcripts, machine learning algorithms trained on structured clinical data, and decision support tools that synthesise multiple information sources all warrant investigation, provided such technologies are validated against appropriate gold standards and implemented with attention to equity and safety considerations.

### 4.7 Conclusions

This validation study provides preliminary evidence that systematic clinical assessment of DSM-5 diagnostic criteria may represent a feasible, safe, and efficient approach to screening and triage for adult ADHD referrals. By achieving perfect sensitivity in this cohort whilst permitting two-fifths of referrals to be triaged to alternative pathways, the criterion-based pathway addresses important challenges facing services managing high ADHD assessment demand by offering a potential solution for ensuring no genuine cases are inappropriately excluded from assessment whilst efficiently allocating specialist resources to those most likely to benefit from it. The perfect negative predictive value confirms that screening decisions distinguished people requiring neurodevelopmental assessment from those whose difficulties reflected alternative explanations in this sample, facilitating appropriate care for all referrals through differentiated pathways. These findings require replication in adequately powered multi-site studies before firm conclusions regarding pathway safety can be drawn.

These findings strengthen the preliminary evidence supporting models in which clinical pathways are redirected from secondary to primary care. By demonstrating that trained non-specialist clinicians can safely conduct initial screening whilst specialists concentrate on comprehensive assessments and complex diagnostic cases, the model offers pragmatic responses to workforce pressures and capacity constraints provided it is validated for safety and supported by adequate training and quality assurance. The criterion-based screening framework also creates a foundation for future developments, including technological enhancements, multi-modal assessment strategies, and differentiated pathways tailored to case complexity.

The increasing recognition of adult ADHD, driven by rising public awareness and improved understanding of neurodevelopmental presentations across the lifespan, presents both opportunities and challenges for mental health services. Opportunities include earlier identification of people who could benefit from evidence-based interventions, reduction of functional impairment and adverse outcomes associated with unrecognised ADHD, and better understanding of neurodevelopmental conditions in adulthood. Challenges include managing escalating demand within constrained resources, distinguishing genuine neurodevelopmental presentations from attention difficulties secondary to other conditions, and ensuring equitable access to assessment for diverse populations. Criterion-based screening pathways, validated to ensure safety and efficiency, represent one component of quality service responses that balance these competing considerations whilst prioritising appropriate care for all people seeking assessment.

The findings of this study indicate that the criterion-based screening model offers preliminary evidence of a level of safety and operational efficiency that compares favourably with traditional self-report instruments or digital assessment tools. Whilst digital continuous performance tests and self-rating scales such as the ASRS are frequently used for their high sensitivity, they often lack the discriminant validity required to safely exclude people in complex clinical populations with high psychiatric comorbidity. Consequently, most conventional screening pathways result in nearly all referred people proceeding to a full diagnostic assessment to avoid false-negative outcomes. In contrast, the Comprehensive ADHD Screening Questionnaire (CASQ) enabled 39.6% of referrals to be safely screened out whilst maintaining a sensitivity of 100.0% and an infinite Number Needed to Harm in this cohort.

Our findings support the need for continued development, validation, and implementation of evidence-based screening approaches that move beyond symptom checklists to systematic examination of diagnostic requirements, thereby facilitating timely access to appropriate care for adults with suspected ADHD.

## Data Availability

The raw data supporting the conclusions of this article will be made available by the authors, without undue reservation.
